# The Patients' Rights

**Published:** 1909-05-22

**Authors:** 


### Nursing Administration.

### THE PATIENTS' RIGHTS.

Logically the patients' position in hospitals and homes is a very strong one. The appeal for funds is made on their behalf. The whole establishment has no other *raison d'être* in the eyes of subscribers but them. The money is given expressly to minister to their wants, and those who give it are, generally speaking, extremely indifferent to the advance of science, the success of a medical school, or the status of a training school for nurses. Yet it is indisputable that a certain school of nurses regard the theory of patients' rights with some impatience. Of course, they would admit that broken legs must be properly mended. The healing process helps to train a probationer, to instruct a student. But the owner of the leg is a nuisance. They do not say this crudely. But they act it, and even put the thought into words in conversing of their patients. The manner engendered by this tone of thought is intensified when patients are not birds of passage, presenting each some fresh matter for observation and professional interest, but are chronic or incurable. Their idiosyncrasies are then too often regarded with a disfavour of which every patient soon becomes aware, and when the institution is largely staffed by untrained or partly trained people, the results are inhumanity, none the less shameful because directed against hearts and minds, seldom manifested in open bodily neglect or cruelty. Before trained nurses were introduced into incurable homes, some of these costly establishments were the scenes of much preventable unhappiness. Discipline was overdone. The attendants assumed a harsh control, and the art of managing fretful sufferers was entirely ignored. In spite of expensive equipment, palatial accommodation, and the efforts of benevolent visitors bent on alleviating suffering, the incurable were often needlessly worried and humiliated, and their comfort was continually put aside at the convenience of those who were appointed to relieve them. Then came a reaction. It began to be felt that patients were entitled to that consideration and respect without which life is intolerable wherever it is lived, and the theory that they ought to be constantly simmering with gratitude while maintained in subservience by sour and overtaxed officials, vanished.

But is there not some danger that in the eagerness to promote contentment the managers in at least one of these establishments are setting to work injudiciously? They appear to conceive that it is possible to maintain a reign of tender humanity towards the patients by means of ceaseless vigilance from without. There never was a more fatal mistake. Difficult of accomplishment, even when surveillance relates to one patient under a nurse at home, it is strangely impracticable when it is attempted over officials in an institution. The spirit of humanity which takes every patient, and enfolds them in a mantle of friendly care, can only be set stirring through the agency of the matron, and, if she is to produce it, those who appoint her must give her full powers. It is not a particularly easy thing to produce this atmosphere among hundreds of sufferers, each one of whom is naturally disposed to centre round self. First and most essential is it that the matron have herself a true vocation, leading her to behold in the wasted bodies and too often maimed minds of her charges the image of her Master—faint it may be, yet never effaced. Unless she is herself reverent in her attitude towards suffering, very pitying in her attitude towards the defects in character occasionally induced by defects in bodily powers, she will labour in vain to make her household contented. In the difficult but very beautiful work which has to be accomplished among the incurable, the matron must lead the way. She cannot hope to stand outside and produce by discipline among others a tone lacking in herself. But when the mind is attuned, it is but the first step. There is still all to do before the right ideals can be translated into act.

And this we believe is where the managers of these homes go astray. They underrate the enormous practical difficulties in the way of nursing incurables and the heavy strain on the nurses whose days are spent in the performance of somewhat depressing routine duties among those whose condition admits of little except alleviation. Nothing but exact discipline can maintain a home of this description in good order, and unless the matron has entire control exact discipline is not to be had. The policy of encouraging complaints to outside authorities may to the unexperienced appear a short cut to securing the comfort of the patients. In reality, for every omission corrected by this means a loophole is made for far more serious abuses. Once loosen the matron's responsibility by ever so little and a rent is made in the fabric which will assuredly wreck the tone of the whole establishment. The patients ought to be able to rely on their matron as their advocate and best friend. They ought to understand that she is working to her utmost to procure them skilled nursing and unfailing attention. They are capable of understanding that with all her care occasional misadventures will occur, that not all nurses are absolutely perfect, and that when the machine is working at its best there is always something to put up with. But when once the right tone of confidence is established, that sensation as of a family party which has long been such a delightful feature of the Middlesex Hospital (to name but one institution where incurable wards are managed to perfection), it is amazing what generous consideration will be shown by the patients towards their nurses. The highest gift which can be bestowed on sufferers is the gift of conscious self-control, of humorous acceptance of unavoidable contretemps, and desire to spare the labourers on their behalf. All this is ruined by a system which perpetually seeks occasion against those who serve the sick. The relation between nurse and patient is too delicate to bear the interference of an outside authority. It is only when the patients begin to forget the existence of "wrongs" that their rights can be considered secure.

